# 2,6-Di­fluoro-*N*-(prop-2-yn­yl)benzamide

**DOI:** 10.1107/S1600536813021120

**Published:** 2013-08-17

**Authors:** Zahid Hussain, Ejaz Hussain, Hina Siddiqui, M. Iqbal Choudhary, Sammer Yousuf

**Affiliations:** a H.E.J. Research Institute of Chemistry, International Center for Chemical and Biological Sciences, University of Karachi, Karachi 75270, Pakistan; b Department of Biochemistry, Faculty of Science, King Abdulaziz University, Jaddhah, Saudi Arabia

## Abstract

In the mol­ecule of the title di­fluoro­benzamide derivative, C_10_H_7_F_2_NO, the angle formed by the least-squares mean line through the prop-2-ynyl group [maximum deviation = 0.011 (3) Å] and the normal to the benzene ring is 59.03 (7)°. In the crystal, mol­ecules are linked *via* N—H⋯O and C—H⋯F hydrogen bonds into layers parallel to the *ac* plane.

## Related literature

For the biological activity of di­fluoro­benzamide derivatives, see: Chang *et al.* (2002 ▶); Kees *et al.* (1989 ▶); Ragavan *et al.* (2010 ▶); Carmellino *et al.* (1994 ▶); Rauko *et al.* (2001 ▶). For the crystal structure of a related compound, see: Fun *et al.* (2010 ▶).
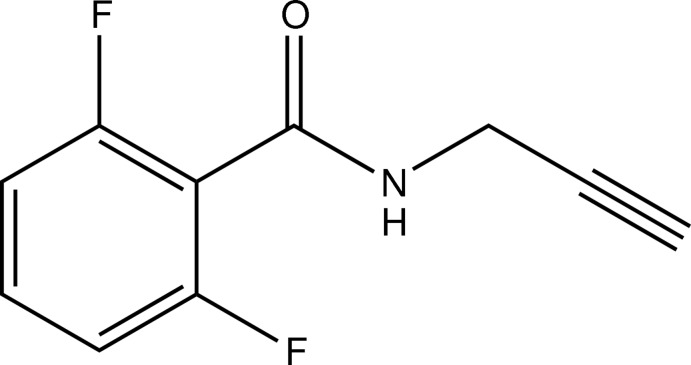


## Experimental

### 

#### Crystal data

C_10_H_7_F_2_NO
*M* *_r_* = 195.17
Monoclinic, 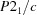

*a* = 5.0479 (8) Å
*b* = 19.738 (3) Å
*c* = 9.2428 (15) Å
β = 91.432 (4)°
*V* = 920.6 (3) Å^3^
*Z* = 4
Mo *K*α radiation
μ = 0.12 mm^−1^
*T* = 273 K
0.38 × 0.17 × 0.10 mm

#### Data collection

Bruker SMART APEX CCD diffractometer
5388 measured reflections
1669 independent reflections
1283 reflections with *I* > 2σ(*I*)
*R*_int_ = 0.022

#### Refinement

*R*[*F* ^2^ > 2σ(*F* ^2^)] = 0.040
*wR*(*F* ^2^) = 0.099
*S* = 1.03
1669 reflections
135 parameters
H atoms treated by a mixture of independent and constrained refinement
Δρ_max_ = 0.13 e Å^−3^
Δρ_min_ = −0.15 e Å^−3^

### 

Data collection: *SMART* (Bruker, 2000 ▶); cell refinement: *SAINT* (Bruker, 2000 ▶); data reduction: *SAINT*; program(s) used to solve structure: *SHELXS97* (Sheldrick, 2008 ▶); program(s) used to refine structure: *SHELXL97* (Sheldrick, 2008 ▶); molecular graphics: *SHELXTL* (Sheldrick, 2008 ▶); software used to prepare material for publication: *SHELXTL*.

## Supplementary Material

Crystal structure: contains datablock(s) global, I. DOI: 10.1107/S1600536813021120/rz5082sup1.cif

Structure factors: contains datablock(s) I. DOI: 10.1107/S1600536813021120/rz5082Isup2.hkl

Supplementary material file. DOI: 10.1107/S1600536813021120/rz5082Isup3.cml

Additional supplementary materials:  crystallographic information; 3D view; checkCIF report

## Figures and Tables

**Table 1:** Hydrogen-bond geometry (Å, °)

*D*—H⋯*A*	*D*—H	H⋯*A*	*D*⋯*A*	*D*—H⋯*A*
N1—H1⋯O1^i^	0.83 (2)	2.10 (2)	2.8387 (19)	147.4 (17)
C2—H2*A*⋯F2^ii^	0.93	2.49	3.394 (2)	164

Symmetry codes: (i) 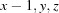; (ii) 
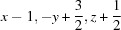
.

**Fig. 1.**
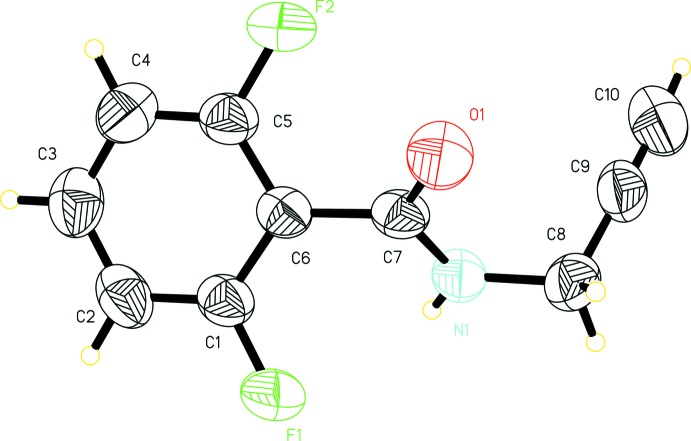
The molecular structure of the title compound, showing displacement ellipsoids at the 50% probability level.

**Fig. 2.**
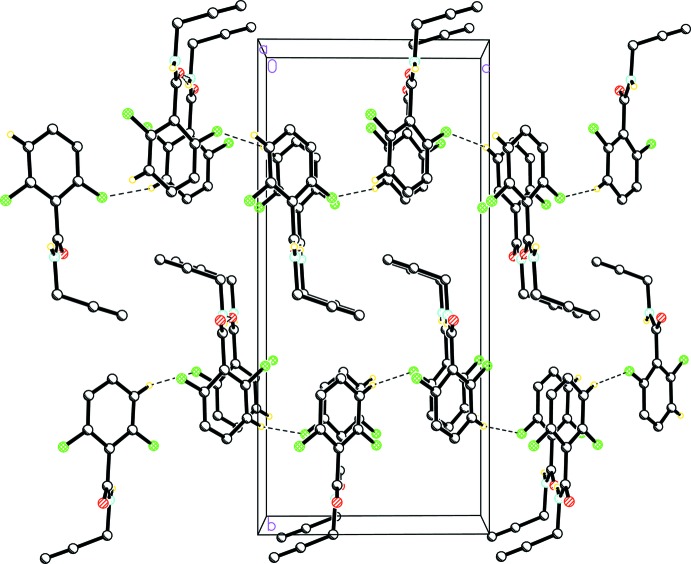
Packing diagram of the title compound showing intermolecular hydrogen bonding as dashed lines.

## supplementary crystallographic information

### 1. Comment

Some difluorobenzamide derivatives are known to have excellent antiviral and antiproliferation activities (Chang *et al.*, 2002). They are also well known for their anti-diabetic (Kees *et al.*, 1989), anti-fungal (Carmellino *et al.*, 1994), anti-bacterial (Ragavan *et al.*, 2010) and anti-cancer (Rauko *et al.*, 2001) properties.

The structure of the title fluorinated benzamide derivative (Fig. 1) is distinctly similar to that of the previously reported compound *N*-(4-cyanophenyl)-2,6-difluorobenzamide (Fun *et al.*, 2010), with the difference that the *N*-(4-cyanophenyl) moiety is replaced by a prop-2-ynyl chain (C8–C10). The observed distance for the C9—C10 acetylene bond is 1.162 (3) Å. The angle between the least-squares mean line through the prop-2-ynyl group (maximum deviation 0.011 (3) Å for atom C9) and the normal to the benzene ring is 59.03 (7)°. The molecule has no prominent intramolecular non-covalent interactions. In the crystal, molecules are linked *via* C—H···F (Fig. 2) and N—H···O hydrogen bonds (Table 1) to form layers parallel to the *ac* plane. No π···π stacking interactions are observed.

### 2. Experimental

Prop-2-yn-1-amine (36.3 mmol, 1.0 eq) was dissolved in dichloromethane (20 mL) in a round bottom flask and kept at 0 °C. Diisopropylethylamine (DIPEA) (145 mmol, 4.0 eq) and 2,6-diflurobenzoyl chloride (54.4 mmol, 1.5 eq) were then added and the mixture stirred for 1.5 h. Progress of the reaction was monitored by thin layer chromatography. On completion of the reaction the mixture was dissolved in water and extracted with diethyl ether (2 × 25 mL). The organic layer was dried with anhydrous Na_2_SO_4_ and concentrated to obtain a crude gummy product. The crude product was finally purified by flash column chromatography by using EtOAc/hexane (3:7 *v*/*v*) as eluent to afford the title compound in 77% yield. Crystals suitable for X-ray analysis were obtained by slow evaporation of an ethanol solution.

### 3. Refinement

The amide and acetylenic H atoms were located in a difference Fourier map and refined freely. All other H atoms were placed at calculated positions and refined as riding, with C—H = 0.93–0.97 Å and with *U*_iso_(H) = 1.2*U*_eq_(C).

### Crystal data

**Table:**

C_10_H_7_F_2_NO	*F*(000) = 400
*M**_r_* = 195.17	*D*_x_ = 1.408 Mg m^−^^3^
Monoclinic, *P*2_1_/*c*	Mo *K*α radiation, λ = 0.71073 Å
Hall symbol: -P 2ybc	Cell parameters from 1280 reflections
*a* = 5.0479 (8) Å	θ = 2.4–22.9°
*b* = 19.738 (3) Å	µ = 0.12 mm^−^^1^
*c* = 9.2428 (15) Å	*T* = 273 K
β = 91.432 (4)°	Block, colourless
*V* = 920.6 (3) Å^3^	0.38 × 0.17 × 0.10 mm
*Z* = 4	

### Data collection

**Table:**

Bruker SMART APEX CCD diffractometer	1283 reflections with *I* > 2σ(*I*)
Radiation source: fine-focus sealed tube	*R*_int_ = 0.022
Graphite monochromator	θ_max_ = 25.5°, θ_min_ = 2.1°
phi and ω scans	*h* = −6→6
5388 measured reflections	*k* = −23→22
1669 independent reflections	*l* = −10→11

### Refinement

**Table:**

Refinement on *F*^2^	Primary atom site location: structure-invariant direct methods
Least-squares matrix: full	Secondary atom site location: difference Fourier map
*R*[*F*^2^ > 2σ(*F*^2^)] = 0.040	Hydrogen site location: inferred from neighbouring sites
*wR*(*F*^2^) = 0.099	H atoms treated by a mixture of independent and constrained refinement
*S* = 1.03	*w* = 1/[σ^2^(*F*_o_^2^) + (0.0397*P*)^2^ + 0.172*P*] where *P* = (*F*_o_^2^ + 2*F*_c_^2^)/3
1669 reflections	(Δ/σ)_max_ < 0.001
135 parameters	Δρ_max_ = 0.13 e Å^−^^3^
0 restraints	Δρ_min_ = −0.15 e Å^−^^3^

### Special details

**Table:**

Geometry. All e.s.d.'s (except the e.s.d. in the dihedral angle between two l.s. planes) are estimated using the full covariance matrix. The cell e.s.d.'s are taken into account individually in the estimation of e.s.d.'s in distances, angles and torsion angles; correlations between e.s.d.'s in cell parameters are only used when they are defined by crystal symmetry. An approximate (isotropic) treatment of cell e.s.d.'s is used for estimating e.s.d.'s involving l.s. planes.
Refinement. Refinement of *F*^2^ against ALL reflections. The weighted *R*-factor *wR* and goodness of fit *S* are based on *F*^2^, conventional *R*-factors *R* are based on *F*, with *F* set to zero for negative *F*^2^. The threshold expression of *F*^2^ > σ(*F*^2^) is used only for calculating *R*-factors(gt) *etc*. and is not relevant to the choice of reflections for refinement. *R*-factors based on *F*^2^ are statistically about twice as large as those based on *F*, and *R*- factors based on ALL data will be even larger.

### Fractional atomic coordinates and isotropic or equivalent isotropic displacement parameters (Å^2^)

**Table:**

	*x*	*y*	*z*	*U*_iso_*/*U*_eq_	
F1	0.4706 (2)	0.66642 (6)	0.01184 (13)	0.0784 (4)	
F2	1.1590 (2)	0.68247 (6)	−0.31627 (13)	0.0808 (4)	
O1	1.0617 (2)	0.56728 (6)	−0.15774 (16)	0.0689 (4)	
N1	0.6219 (3)	0.55850 (7)	−0.17077 (17)	0.0520 (4)	
C1	0.6337 (3)	0.70378 (9)	−0.06950 (19)	0.0533 (5)	
C2	0.6133 (4)	0.77291 (10)	−0.0635 (2)	0.0670 (6)	
H2A	0.4884	0.7934	−0.0055	0.080*	
C3	0.7807 (4)	0.81150 (10)	−0.1445 (2)	0.0690 (6)	
H3A	0.7685	0.8585	−0.1419	0.083*	
C4	0.9655 (4)	0.78117 (10)	−0.2290 (2)	0.0664 (5)	
H4A	1.0802	0.8071	−0.2835	0.080*	
C5	0.9778 (3)	0.71209 (9)	−0.2314 (2)	0.0546 (5)	
C6	0.8147 (3)	0.66992 (8)	−0.15372 (17)	0.0453 (4)	
C7	0.8430 (3)	0.59431 (9)	−0.16021 (17)	0.0475 (4)	
C8	0.6276 (4)	0.48497 (9)	−0.1809 (2)	0.0607 (5)	
H8A	0.4535	0.4673	−0.1599	0.073*	
H8B	0.7519	0.4674	−0.1085	0.073*	
C9	0.7041 (4)	0.46128 (9)	−0.3233 (2)	0.0621 (5)	
C10	0.7642 (5)	0.44382 (12)	−0.4380 (3)	0.0880 (7)	
H1	0.475 (4)	0.5775 (9)	−0.1715 (19)	0.061 (6)*	
H2	0.817 (5)	0.4327 (13)	−0.524 (3)	0.120 (10)*	

### Atomic displacement parameters (Å^2^)

**Table:**

	*U*^11^	*U*^22^	*U*^33^	*U*^12^	*U*^13^	*U*^23^
F1	0.0691 (7)	0.0792 (8)	0.0889 (9)	−0.0091 (6)	0.0395 (6)	−0.0161 (6)
F2	0.0727 (7)	0.0810 (8)	0.0907 (9)	−0.0041 (6)	0.0418 (7)	−0.0052 (6)
O1	0.0315 (6)	0.0655 (8)	0.1096 (11)	0.0057 (5)	0.0013 (6)	−0.0028 (7)
N1	0.0316 (7)	0.0521 (9)	0.0726 (11)	0.0022 (6)	0.0071 (7)	−0.0047 (7)
C1	0.0412 (9)	0.0628 (11)	0.0559 (11)	−0.0019 (8)	0.0054 (8)	−0.0104 (9)
C2	0.0543 (10)	0.0674 (13)	0.0794 (14)	0.0088 (9)	0.0033 (10)	−0.0230 (10)
C3	0.0648 (12)	0.0532 (11)	0.0885 (16)	0.0037 (9)	−0.0073 (11)	−0.0048 (10)
C4	0.0604 (11)	0.0634 (12)	0.0753 (14)	−0.0065 (9)	0.0039 (10)	0.0069 (10)
C5	0.0432 (9)	0.0629 (11)	0.0579 (11)	−0.0003 (8)	0.0070 (8)	−0.0048 (9)
C6	0.0320 (8)	0.0553 (10)	0.0484 (10)	0.0000 (7)	−0.0021 (7)	−0.0053 (8)
C7	0.0328 (8)	0.0571 (10)	0.0527 (10)	0.0014 (7)	0.0052 (7)	−0.0023 (8)
C8	0.0503 (10)	0.0529 (11)	0.0794 (14)	−0.0025 (8)	0.0094 (9)	0.0042 (9)
C9	0.0579 (11)	0.0451 (10)	0.0835 (16)	0.0055 (8)	0.0048 (11)	−0.0031 (10)
C10	0.1032 (19)	0.0689 (15)	0.092 (2)	0.0121 (12)	0.0132 (16)	−0.0128 (14)

### Geometric parameters (Å, º)

**Table:**

F1—C1	1.3482 (19)	C3—H3A	0.9300
F2—C5	1.3530 (18)	C4—C5	1.365 (3)
O1—C7	1.2256 (17)	C4—H4A	0.9300
N1—C7	1.323 (2)	C5—C6	1.384 (2)
N1—C8	1.455 (2)	C6—C7	1.501 (2)
N1—H1	0.830 (19)	C8—C9	1.458 (3)
C1—C2	1.370 (3)	C8—H8A	0.9700
C1—C6	1.387 (2)	C8—H8B	0.9700
C2—C3	1.374 (3)	C9—C10	1.162 (3)
C2—H2A	0.9300	C10—H2	0.87 (3)
C3—C4	1.369 (3)		
			
C7—N1—C8	121.31 (15)	F2—C5—C6	117.42 (16)
C7—N1—H1	120.7 (13)	C4—C5—C6	124.38 (16)
C8—N1—H1	118.0 (13)	C5—C6—C1	114.22 (16)
F1—C1—C2	118.34 (15)	C5—C6—C7	121.27 (14)
F1—C1—C6	118.01 (16)	C1—C6—C7	124.49 (15)
C2—C1—C6	123.65 (17)	O1—C7—N1	121.78 (16)
C1—C2—C3	118.85 (17)	O1—C7—C6	121.24 (14)
C1—C2—H2A	120.6	N1—C7—C6	116.97 (13)
C3—C2—H2A	120.6	N1—C8—C9	112.60 (15)
C4—C3—C2	120.37 (18)	N1—C8—H8A	109.1
C4—C3—H3A	119.8	C9—C8—H8A	109.1
C2—C3—H3A	119.8	N1—C8—H8B	109.1
C5—C4—C3	118.53 (18)	C9—C8—H8B	109.1
C5—C4—H4A	120.7	H8A—C8—H8B	107.8
C3—C4—H4A	120.7	C10—C9—C8	178.5 (2)
F2—C5—C4	118.19 (16)	C9—C10—H2	176.4 (19)
			
F1—C1—C2—C3	179.19 (17)	C2—C1—C6—C5	0.6 (3)
C6—C1—C2—C3	−0.2 (3)	F1—C1—C6—C7	−0.3 (2)
C1—C2—C3—C4	−0.4 (3)	C2—C1—C6—C7	179.05 (18)
C2—C3—C4—C5	0.4 (3)	C8—N1—C7—O1	−0.4 (3)
C3—C4—C5—F2	179.33 (17)	C8—N1—C7—C6	178.68 (15)
C3—C4—C5—C6	0.1 (3)	C5—C6—C7—O1	41.8 (2)
F2—C5—C6—C1	−179.83 (15)	C1—C6—C7—O1	−136.53 (18)
C4—C5—C6—C1	−0.6 (3)	C5—C6—C7—N1	−137.26 (17)
F2—C5—C6—C7	1.7 (2)	C1—C6—C7—N1	44.4 (2)
C4—C5—C6—C7	−179.07 (18)	C7—N1—C8—C9	−75.0 (2)
F1—C1—C6—C5	−178.74 (15)		

### Hydrogen-bond geometry (Å, º)

**Table:**

*D*—H···*A*	*D*—H	H···*A*	*D*···*A*	*D*—H···*A*
N1—H1···O1^i^	0.83 (2)	2.10 (2)	2.8387 (19)	147.4 (17)
C2—H2*A*···F2^ii^	0.93	2.49	3.394 (2)	164

Symmetry codes: (i) *x*−1, *y*, *z*; (ii) *x*−1, −*y*+3/2, *z*+1/2.
